# Unraveling the genetic basis of general combining ability in CIMMYT elite bread wheat germplasm: implications for breeding strategies optimization

**DOI:** 10.3389/fpls.2025.1675993

**Published:** 2025-10-17

**Authors:** José I. Saavedra-Ávila, Guillermo S. Gerard, Salvatore Esposito, Velu Govindan, Julio Huerta-Espino, Zerihun Tadesse, Susanne Dreisigacker, Carolina Saint Pierre, Angela Pacheco, Fernando Toledo, Keith A. Gardner, Leonardo Crespo-Herrera, José Crossa, Paolo Vitale

**Affiliations:** ^1^ Departamento de Genética, Colegio de Postgraduados, Estado de México, Montecillo, Mexico; ^2^ International Maize and Wheat Improvement Center (CIMMYT), Texcoco, Estado de México, Mexico; ^3^ Institute of Bioscience and BioResources, National Research Council, Portici, Italy; ^4^ Campo Experimental Valle de México INIFAP, Chapingo, Estado de México, Mexico

**Keywords:** wheat breeding, general combining ability, GWAS, genomic prediction, cross-efficiency

## Abstract

In wheat breeding programs, several hundred crosses are performed annually, but only individuals from a few families advance to the final stages of the breeding pipelines. Therefore, a deeper understanding of the general combining ability (GCA) of wheat genotypes might enhance the breeding efficiency in selecting parents. For this reason, we tested the performance of the offspring of ~1200 parental elite lines. Using a genome-wide association study (GWAS), gene ontology (GO) analysis, and genomic prediction (GP), our objectives were to i) identify marker-trait associates (MTAs) and candidate genes, ii) assess temporal allele frequency dynamics of identified MTAs, and iii) estimate prediction accuracy (PA) for key traits: Progeny Number per-Cross (PNC), grain yield (GY), and a combined index incorporating these traits (“index”). Our findings revealed a total of 13 MTAs: eight for GY, four for the “index”, and one for PNC. The GO analysis highlighted several genes involved in hydrogen peroxide metabolism and catabolism processes (H_2_O_2_), reactive oxygen species, response to oxidative stress, cell wall biogenesis, the metabolic process of modified amino acids at the cellular level, and glutathione metabolic process for the studied traits. Notably, allele frequency analysis over time indicated that most MTAs are under positive selection, likely reflecting indirect breeder-driven selection. The highest PA was reached by using the reproducing kernel Hilbert space (RKHS) model for the trait GY (0.34). The identification of MTAs for PNC and GY provided insight into the biological pathways underpinning combining ability and demonstrated the potential for predicting the ability of the genotypes to be crossed. These findings might contribute to the optimization crossing strategy saving costs and increasing the breeding program efficiency.

## Graphical Abstract

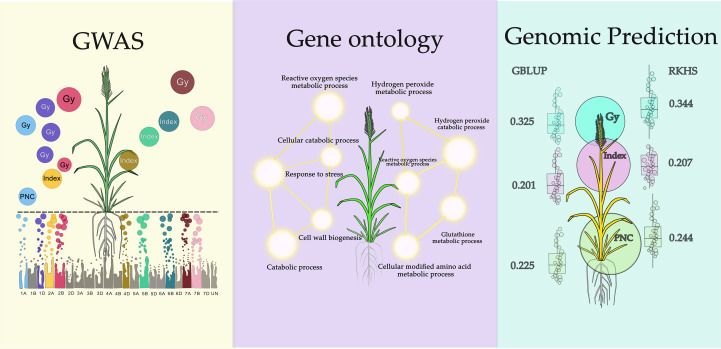

## 1 Introduction

Wheat is one of the most important staple crop globally, serving as a primary source of nutrients for approximately 40% of the world’s population (Giraldo et al., 2019; Reynolds and Braun, 2022). It is also the most widely cultivated crop worldwide, growing on over 217 million hectares of land (Erenstein et al., 2022). The breeding of new wheat varieties involves generating genetic variations through controlled crosses, self-fertilization, advanced generation selection, field trials, and several quality analyses, which entails a considerable investment of time (10 to 15 years) and resources (Haile et al., 2021).

Combining ability (CA) is the ability of the plants to combine with each other in order to transmit their desirable traits to their offspring; thus, by crossing one line with many others, it is possible to observe its average performance in all its crosses and highlight that the general combining ability (GCA) is described as the mean performance of a genotype across multiple crosses. By contrast, the specific combining ability (SCA) is characterized as deviations from the expected performance in certain combinations, either exceeding or falling short of the average performance of the parental inbred lines (Fasahat et al., 2016; Sprague and Tatum, 1942). CA has been widely studied in several crops, such as maize (Dermail et al., 2023; Ertiro et al., 2013; Makumbi et al., 2011; Ravikesavan et al., 2020; Run et al., 2013), cotton (Aishwarya et al., 2025; Anandan, 2010; Zeng and Pettigrew, 2015), sunflower (Habib et al., 2021; Ortis et al., 2005; Volotovich et al 2008), alfalfa (Bhandari et al., 2007; Lawati et al., 2010), and rice (Azad et al., 2022; Shukla and Pandey, 2008; Verma and Srivastava, 2004). In wheat (*Triticum aestivum* L.), the presence of GCA has been statistically demonstrated using a 7 × 7 diallel set of bread wheat, also highlighting the potential to identify superior general combiners (Kumar et al., 2011). Furthermore, studies have shown that utilizing the GCA for parent selection can be an effective strategy to enhance wheat breeding programs (Gowda et al., 2012). In addition, the assessment of GCA and SCA in wheat germplasm from Pakistan has successfully identified superior combiners for grain yield and related traits (Iqbal, 2007). Despite these advancements, wheat breeding programs continue to produce hundreds of inefficient crosses each year. Understanding the genetic and molecular basis of GCA and SCA could facilitate efficient parent selection and crossing, accelerating the production of elite cultivars.

Genome-wide association studies (GWAS) aim to identify associations between single-nucleotide polymorphisms (SNPs) and phenotypic traits of interest, such as complex characters like yield. This allows for accelerated crop improvement through molecular marker-assisted and allele stacking selection. GWAS or quantitative trait loci (QTL) analyses have been performed in wheat for a variety of traits, such as grain yield and yield components (Eltaher et al., 2021; Li et al., 2019), phenology (Zhang et al., 2018), disease resistance (Singh et al., 2020; Tessmann et al., 2019), morphological traits (Sheoran et al., 2019; Vitale et al., 2021), and quality traits (Tadesse et al., 2015; Yang et al., 2020). Studies in maize have shown that the identification of key loci for GCA by GWAS could accelerate breeding and the selection of elite parents for the creation of hybrids (Liu et al., 2021; Lu et al., 2020). Association studies related to CA have been carried out in recent years, mainly in crops such as rice, corn, and cotton, in the case of rice, it was found that the accumulation of superior GCA and SCA alleles contributes to heterosis and that significant QTLs favor combinatorial ability, which could accelerate the selection of the best parents for the development of hybrids (Chen et al., 2019; Li et al., 2022; Sarfraz et al., 2021).

Additionally, several QTLs have been linked to CA in rice, along with their pleiotropic effects on other agronomic traits (Qu et al., 2012). Notably, Lu et al. (2020) also identified numerous QTLs associated with per se performance and corresponding GCA effects for yield-related traits, which hold potential for improving maize hybrid breeding.

However, for a deeper analysis, it is necessary to complement them with other tools, such as gene ontology (GO) analysis, used to represent biological functions over genes, using a standardized vocabulary (Ashburner et al., 2000; Meng et al., 2009). Indeed, GO analyses have been performed following GWAS studies to investigate CA in rice (Chen et al., 2019; Ullah Zaid et al., 2019).

Genomic prediction (GP) is a technique for estimating phenotypic values from genotypic data, utilizing molecular information from the entire genome. Its use has increased significantly due to the low cost and incorporation of all or most of the markers, making it an essential tool in breeding programs for predicting traits of interest (Bernardo, 2008; Crossa et al., 2017; Meuwissen et al., 2001). GP has been applied for predicting a wide range of traits across several crops, such as wheat (Crossa et al., 2014; Lado et al., 2013), rice (Bartholomé et al., 2022; Labroo et al., 2021), maize (Crossa et al., 2013; Technow et al., 2014) and soybean (Jarquín et al., 2014; Zhang et al., 2016). In corn, GP has been used to predict CA in order to evaluate the performance of lines and hybrids more efficiently (Zhang et al., 2022). Furthermore, the effectiveness of predicting GCA using genomic prediction models has been evaluated. The study also compared GP application with phenotyping methods, concluding that the application of GP is a more effective and efficient approach for predicting the GCA of maize lines and their hybrid performance (Vélez‐Torres et al., 2018). Finally, Werner et al. (2018) evaluated the accuracy of genomic prediction for various agronomic traits in oilseed rape. Their analysis utilized ridge-regression best linear unbiased prediction (BLUP) and three Bayesian alphabet models, considering both GCA and SCA.

The objectives of our study are: 1) to identify the marker traits associations for three traits related to the GCA (GY, “index”, and PNC) using GWAS analysis; 2) to uncover biological pathways or metabolic processes overrepresented among the identified genes through genetic enrichment analysis; 3) assess temporal allele frequency dynamics of identified MTAs over 10 years of data, and 4) to evaluate the genomic prediction accuracy for these traits, enabling practical implementation in wheat breeding programs.

## 2 Materials and methods

### 2.1 Plant material and combining ability-related traits

The phenotypic dataset comprised 1203 CIMMYT (International Maize and Wheat Improvement Center) elite breeding lines used as parents in the CIMMYT bread wheat breeding program crossing block over five years (from the 2013/14 to 2017/18 seasons). The offspring of these breeding lines that reached the grain yield (GY) evaluation stages were tested across multiple years (from 2017/18 to 2022/2023) at the CENEB (Campo Experimental Norman E. Borlaug) research station (27°20′ N, 109°54′ W). The yield trials were conducted following a raised bed planting system, under optimal irrigated conditions (B5IR) (approximately 500 mm of water supplied across five irrigation events) and an optimal sowing date (late November to mid-December). They were arranged in an alpha lattice design with two replicates, using a 4.48 m² plot size and a seeding rate of 120 kg ha^-^¹. At maturity, whole plots were harvested to assess GY. GY was standardized to a moisture content of 12%. Finally, weather parameters including solar flux, temperatures, humidity, precipitation, wind pressure and soil proprieties were downloaded for each crop season from the NASAPOWER website (https://www.nasa.gov/) and shared in the open repository Figshare (https://doi.org/10.6084/m9.figshare.30025069.v1).

### 2.2 Statistical analysis and combining ability

The GY phenotypic performances of the offspring produced by the 1203 parents were analyzed using the lme4 package (Bates et al., 2015) in R software (Team, 2016), using the following statistical model (Equation 1):


(1) $$y_{ijk}=\text{μ}+r_{j}+b_{k\left(j\right)}+g_{i}+{\text{ϵ}}_{ijk}$$

where y_ijk_ is the observed value, where μ is the general mean, $r_{j}$ is the random effects of the replicates (j = 1, …, 3), $g_{i}$ is the random effect of the wheat genotype, assumed to be identically and independently normally distributed (IID) with mean zero and variance $\sigma_{g}^{2}$ , and $b_{k\left(j\right)}\;$ represents the random effects of the incomplete blocks (k = 1, …, 5) nested within replicate, and it is assumed (IID) with mean zero and variance $\sigma_{b}^{2}$ . The term ${\text{ϵ}}_{ijk}$ is a random residual assumed to be IID with mean zero and variance $\sigma_{\text{ϵ}}^{2}$ . Then, we fit the same model but now with $g_{i}$ as fixed effects to estimate adjusted means (Best Linear Unbiased Estimates, BLUEs).

To estimate GCA, the GY of each elite parental line was calculated as the average of its progeny. Before the calculation of the CGA, the GY BLUEs of each progeny were expressed in terms of the cultivar Borlaug 100 (common check across all the GY trials). Therefore (Equation 2),


(2) $$g_{i}={\bar{F}}_{i}-{\bar{F}}_{.}$$

Where $g_{i}$ is the GCA effect of inbred lines *i*; ${\bar{F}}_{i}$ is the average value of the progeny involving the inbred line *i* as parent, and ${\bar{F}}_{.}\;$ is the average value of all progenies.

Additionally, the number of progenies from each elite parental line was tallied and divided by the number of crosses in which the parental line participated (PNC). Finally, GY and PNC were expressed using a scale from 1 to 2 (1 minimum value and 2 maximum value), and they were used to calculate an “index” using the following formula (Equation 3):


(3) index=(GY∗0.6)∗(PNC∗0.4)

Phenotipic dataset was public shared in the open access repository Figshare (https://doi.org/10.6084/m9.figshare.30024511.v1).

### 2.3 Genotyping value

The genotypic data comprised a total of 18,239 SNP markers, generated using the Genotyping-by-Sequencing (GBS) approach. This process was carried out on an Illumina HiSeq2500 sequencer at Kansas State University, following the protocol described by (Poland et al., 2012). Data quality was ensured through meticulous filtering conducted with TASSEL v5.0 software (https://tassel.bitbucket.io) (Bradbury et al., 2007). Markers with a minor allele frequency (MAF) below 5% and those with over 50% missing data were excluded during the initial processing. The filtered HapMap was used to perform GWAS analysis. Subsequently, the HapMap was converted into a numerical matrix, ensuring compatibility with genomic prediction tools. Using the curated marker dataset, a genomic relationship matrix (***G***) was calculated with the AGHmatrix v2.1.4 R package (Amadeu et al., 2023). In addition, linkage disequilibrium (LD) was assessed by calculating the squared correlation coefficient (r²) of allele frequencies for all pairwise SNP combinations within each chromosome using the TASSEL v5.0 software. To examine LD decay, r² values were plotted against the physical distance (in Mb) between marker pairs on each chromosome. Additionally, Kinship matrix and Principal Component Analysis (PCA) were also performed in TASSEL environment. The resulting filtered HapMap was made publicly available on figshare (https://doi.org/10.6084/m9.figshare.29669330.v1).

### 2.4 Genome-wide association study

We conducted the GWAS analysis using, as input, the GCA values, PNC values, and the selection index described in the “Data Analysis and Combining Ability” section, along with the filtered marker dataset outlined above. For the GWAS study, we used R software using the GAPIT v3.0 library (Genome Association and Prediction Integrated Tool) (Lipka et al., 2012); two methods of analysis were used; Bayesian information and Linkage disequilibrium Iteratively Nested Keyway (BLINK) (Huang et al., 2019) and Fixed and random model Circulating Probability Unification (FarmCPU) (Liu et al., 2016). In addition, a principal component parameter (PCA = 3) was incorporated to detect associations between markers and phenotypes (GY, PNC, and “index”). Finally, the significance threshold for the MTAs was identified using a Bonferroni correction α = 0,05 with a p = 5.0561 x 10^-6^ (-log10 p = 5.30). Following the identification of significant MTAs, the favorable allele for each locus was determined by inspecting allele-specific phenotypic distributions via boxplots. The allele associated with the higher average value of the target traitxwas classified as favorable. Based on these classifications, we generated Favorable Allelic Combinations (FACs) for GY and the selection “index” by compiling all observed multi-locus combinations of favorable alleles across the respective sets of MTAs. These FACs were then used to evaluate the additive effect of multiple favorable alleles within genotypes, offering a composite view of their contribution to trait expression.

#### 2.4.1 Candidate genes, gene ontology, and changes in favorable allele frequency

Flanking sequences covering (±)1 Mb of the significant markers from the GWAS analysis results, were analyzed in comparison with the wheat reference genome (IWGSC RefSeq v1.0) (Consortium et al., 2018). For this purpose, the Linux operating system was used, utilizing tools for manipulating genomic data, such as “gawk” (Robbins, 2004) and “bedtools” (Quinlan and Hall, 2010). For the genetic enrichment analysis, all the candidate genes for each trait were taken and analyzed using the tool ShinyGO v0.741, with a significance threshold p-value (FDR) of 0.05 (Ge et al., 2020). Following the identification of significant marker-trait associations, favorable alleles were determined by comparing the mean phenotypic values associated with each allele using boxplots. For each of the 13 MTAs, the allele linked to the more desirable phenotypic performance was classified as favorable. To evaluate changes in favorable allele frequencies over time, we leveraged a historical dataset from the CIMMYT wheat breeding program spanning ten consecutive crop seasons. Specifically, data from ten years of Elite Yield Trials (EYT), covering breeding cycles from 2013–2014 to 2022–2023, were used to monitor the temporal trends in favorable allele frequencies. For a detailed description of the EYT dataset and its structure, refer to Vitale et al. (2025).

### 2.5 Genomic prediction

For GP analysis, the same input data as for GWAS was used, with exception of the numerical SNPs conversion (1, 0, and 2) to meet software requirements. Markers were scaled and centered, and the matrix of genomic relationships was calculated (***G***) proposed by Van Raden (VanRaden, 2008). The five-fold cross-validation process was carried out (CV) 10 times. Prediction accuracy was assessed by correlating the predicted values with the observed phenotypes. The genomic best linear unbiased prediction (GBLUP) model was performed as follows (Equation 4):


(4) y=μ1+Zg+ϵ

where y is the vector of the phenotypes, μ corresponds to the intercept, Z corresponds to the design matrix of random effects, g is the vector of genomic breeding values, and *ϵ* is the vector of random errors. It is also assumed that $g∼\text{N(}0\mathrm{,G}\sigma_{G}^{2}\text{)}$ , where G is the genomic relationship matrix, and $\sigma_{G}^{2}$ is the additive genetic variance (VanRaden, 2008).

Subsequently, RKHS with kernel averaging was applied as follows (Equation 5):


(5) $$y=\mu +\sum_{l=1}^{L}u_{l}+\epsilon$$

Where y , μ , and ϵ have been reported in Equation 4, and $u_{l}∼N\mathrm{(0,}K_{l}{\text{σ}}_{u_{l}}^{2})$ is the additive genetic effect with $K_{l}$ corresponding to the Gaussian reproducing kernel evaluated at *l*th of bandwidth parameters and ${\text{σ}}_{u_{l}}^{2}$ is the additive genetic variance as reported in the package BGLR (Pérez and de Los Campos, 2014). Both GBLUP and RKHS models were run as single strings of 12,000 iterations, of which the first 5,000 were discarded as burnIn in order to ensure that the model had reached convergence. The analysis was carried out using the BGLR library (Bayesian Generalized Linear Regression).

## 3 Results

### 3.1 Population phenotypic analysis

**Figure 1:**
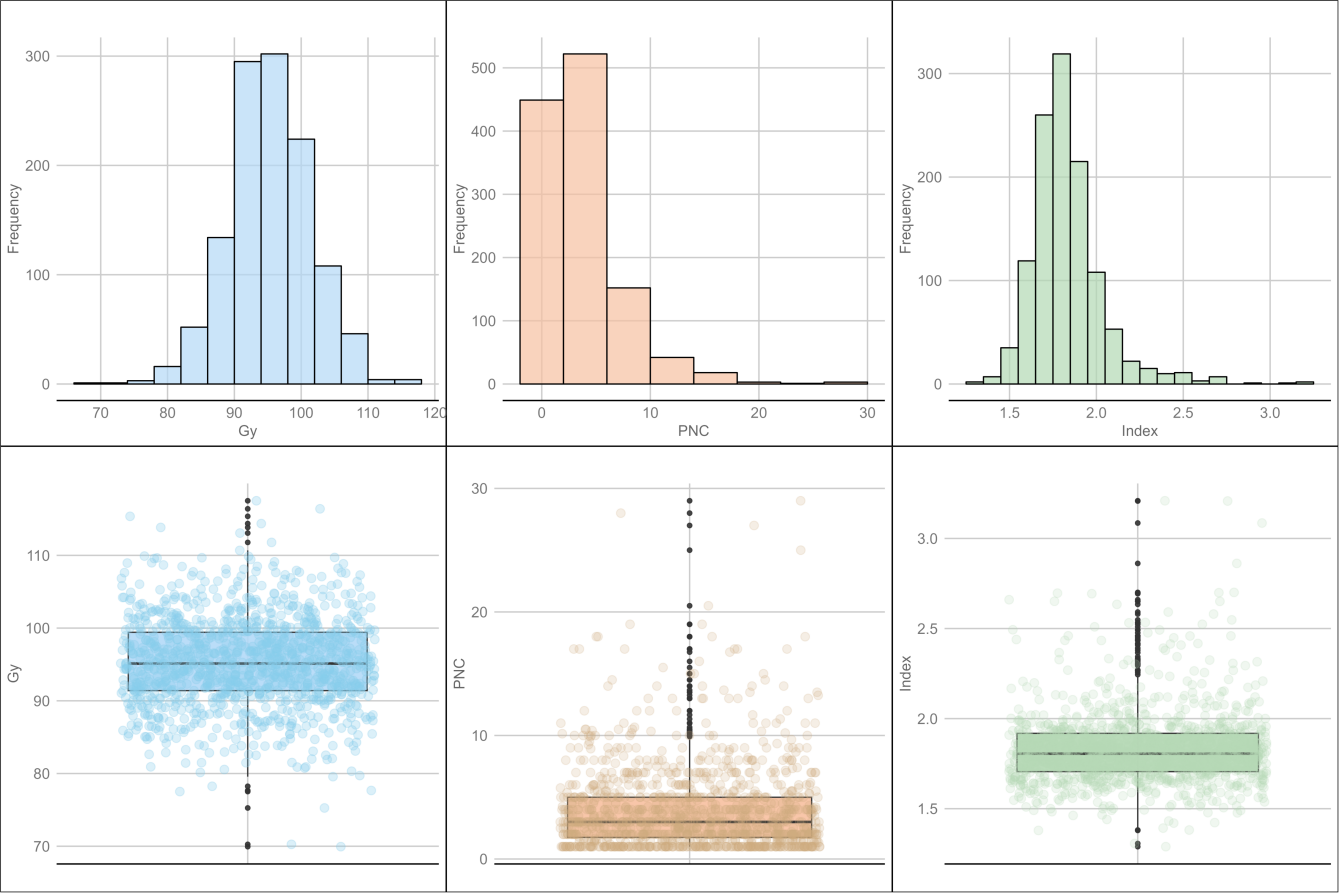
Distribution and box plots for combining ability-related traits: grain yield (GY), Progeny Number per-Cross (PNC), and the “index”.

Descriptive statistics showed variations for all variables. The average grain yield was 95.3%, with a minimum of 70.0% and a maximum of 117.5% of Borlaug 100, and a standard deviation (SD) of 6.2, corresponding to a coefficient of variation (CV) of 6.5%. The “index” variable had values from 1.3 to 3.2, with a mean of 1.8, a CV of 11.3%, and an SD of 0.2. Finally, PNC exhibited an average of 4.0, with minimum values of 1.0 to a maximum of 29.0; its CV was 86.3%, and its SD was 3.5 (**Figure 1**).

### 3.2 Genome-wide association study

**Figure 2:**
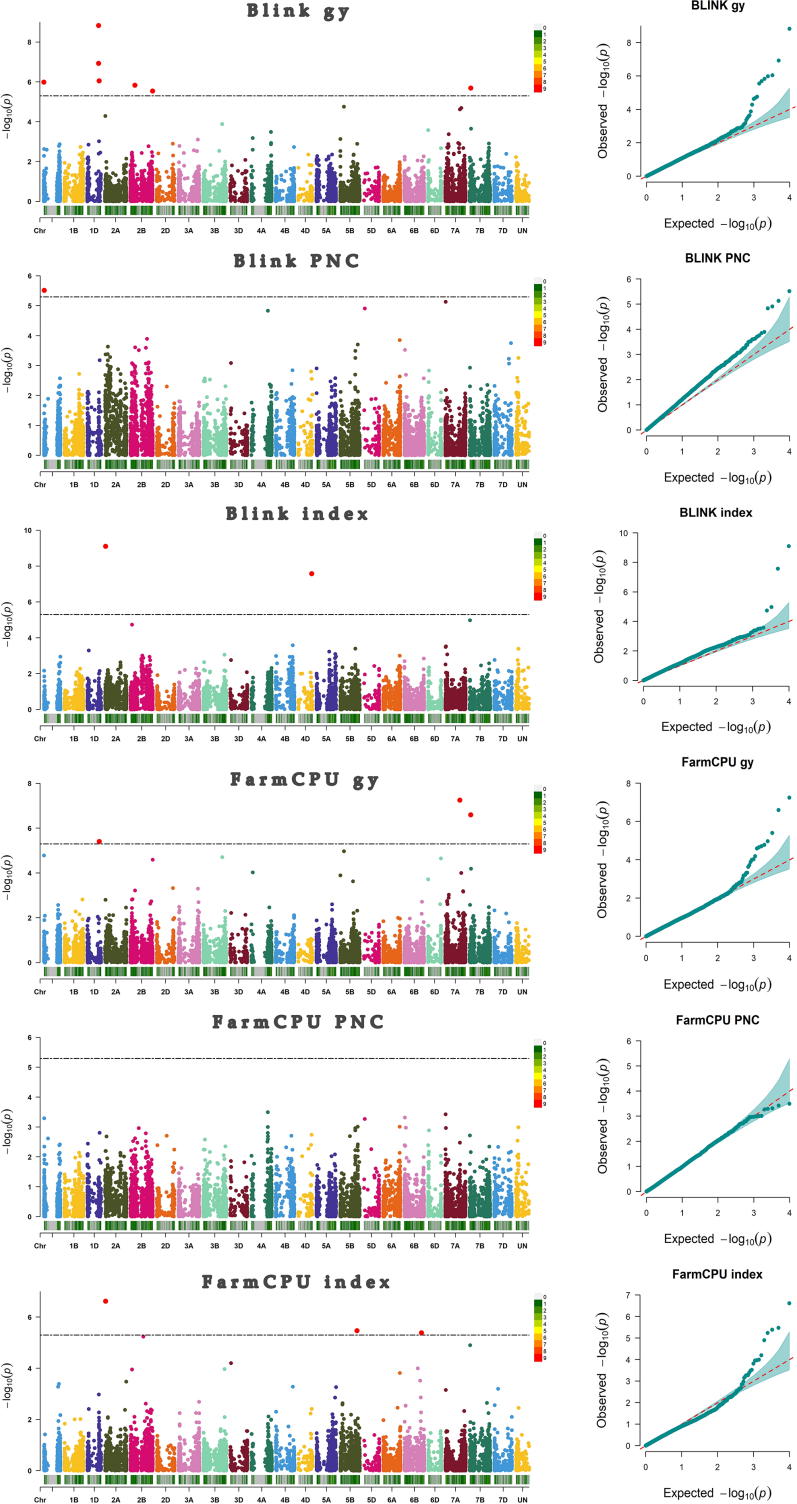
Manhattan and qqplot plots representing markers-traits associations. The red points above the black line (LOD ≥ 5.30) represent the significant markers associated with grain yield (GY), Progeny Number per-Cross (PNC), and the “index”.

After filtering, 1190 genotypes and 9889 markers were identified. **Supplementary Figure S1** displays the distribution of the markers along the three wheat genomes. LD ranged from 1.8 Mb to 15.1 Mb for the chromosome 6D and 2D, respectively (**Supplementary Figure S2**). Kinship and PCA were also reported in **Supplementary Figures S3, S4**, respectively. A total of 13 significant associations between markers and phenotypic traits were detected; the BLINK model identified 62.5%, while FarmCPU identified 37.5% of all associations. (**Figure 2**) We identified eight markers associated with GY, four to “index”, and one associated with PNC. The majority of associations were located on chromosome 1. Two associations were found on chromosome 1A and three on chromosome 1D (5 associations), while chromosomes 2 and 7 showed 3 and 2 associations, respectively. Chromosome 2 had one association on 2A and two on 2B, while chromosome 7 showed an association in 7A and another in 7B. Chromosomes 4D, 5B, and 6B showed only one association each. It is worth mentioning that the BLINK and FarmCPU models identified the same marker on chr 1D at ~432 Mb, on chr 2A at ~15 Mb, and on chr 7B at ~44 Mb. In addition, the *S1A_9565863* marker contributed 2.68% of the phenotypic variation explained (PVE) for the PNC variable (**Table 1**).

**Table 1:** List of significant markers associated with grain yield (GY), Progeny Number per-Cross (PNC), and the “index”.

Number	Traits	SNP	Chr	Pos (Mbp)	Model	LOD	PVE	Ref/Pos (Mpb)
1	GY	S1A_1158042	1A	1.2	BLINK	5.98	3.89	
2	GY	S1D_411724142	1D	411.7	BLINK	6.93	2.98	(488.6–493.0) (Liu et al., 2020a)
3	GY	S1D_412504493	1D	412.5	BLINK	8.83	4.39	(488.6–493.0) (Liu et al., 2020a)
4	GY	S1D_432638693	1D	432.6	BLINK	6.05	3.35	(488.6–493.0) (Liu et al., 2020a)
	GY	S1D_432638693	1D	432.6	FarmCPU	5.4	0	
5	GY	S2B_161419325	2B	161.4	BLINK	5.83	2.25	
6	GY	S2B_800784801	2B	800.8	BLINK	5.54	3.48	(White et al., 2022) (766.2)
7	GY	S7A_533059303	7A	533.1	FarmCPU	7.25	NA	
8	GY	S7B_44882828	7B	44.9	BLINK	5.69	3.75	
	GY	S7B_44882828	7B	44.9	FarmCPU	6.59	3.65	
9	“Index”	S2A_15755581	2A	15.8	BLINK	9.1	5.75	(Liu et al., 2020a) (32.0 –32.9); (Li et al., 2019) (27.3–32.0); (White et al., 2022) (18.6)
	“Index”	S2A_15755581	2A	15.8	FarmCPU	6.62	0.3	
10	“Index”	S4D_481167093	4D	481.2	BLINK	7.57	4.87	(Khan et al., 2022) (465.8)
11	“Index”	S5B_610327124	5B	610.3	FarmCPU	5.47	0	(692.7–700.9) (Li et al., 2019)
12	“Index”	S6B_616781941	6B	616.8	FarmCPU	5.39	0.28	
13	PNC	S1A_9565863	1A	9.6	BLINK	5.51	2.68	

**Figure 3:**
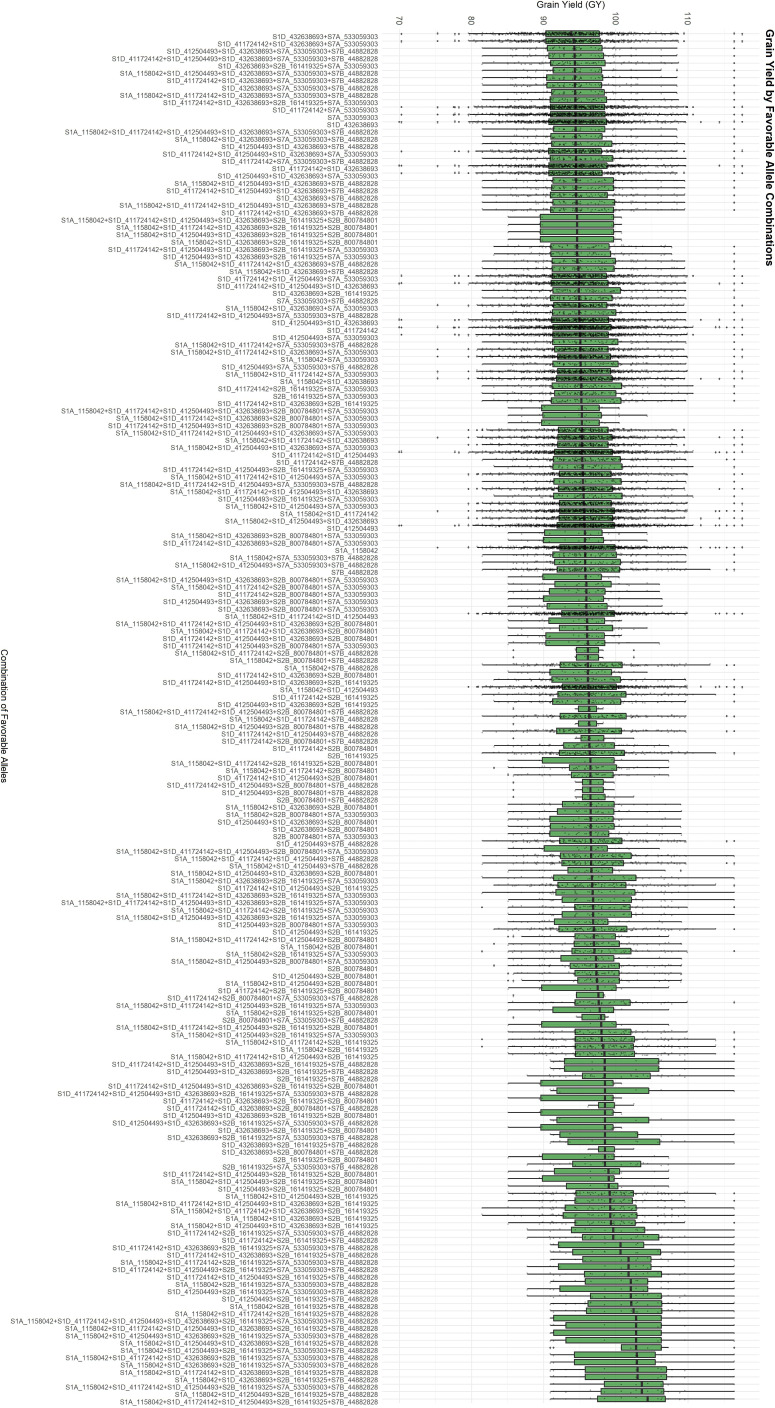
Favorable Allelic Combinations (FACs) based on the eight MTAs associated with grain yield (GY), showing a gradient in mean GY across 187 observed combinations.

**Figure 4:**
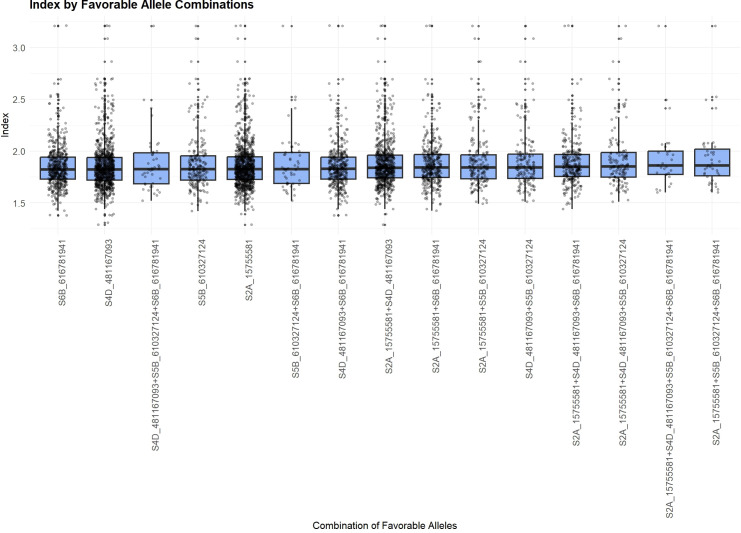
Favorable Allelic Combinations (FACs) based on the four MTAs associated with the “index”, showing limited variation in mean “index” values.

To identify favorable allele combinations, we examined boxplots showing the phenotypic distribution associated with each allele across the 13 significant MTAs and their respective target traits. For each MTA, the allele contributing to superior phenotypic performance was designated as favorable. Based on this, we generated all observed combinations of favorable alleles, referred to as FACs for GY (**Figure 3**) and the “index” (**Figure 4**). FACs were not computed for PNC, as only a single significant MTA was identified for this trait. For GY, we identified 187 unique FACs in the dataset and observed a clear gradient in mean phenotypic performance across combinations. The mean GY ranged from 93.60 to 102.58. The lowest-performing combination was composed of the favorable alleles at markers *S1D_411724142*, *S1D_412504493*, *S1D_432638693*, *S2B_800784801*, and *S7A_533059303*. In contrast, the highest-performing FAC included favorable alleles at *S1A_1158042*, *S1D_411724142*, *S1D_412504493*, *S2B_161419325*, and *S7B_44882828*.

In addition, FACs derived from the four MTAs associated with the “index” did not exhibit a clear trend. Mean “index” values ranged only slightly, from 1.85 to 1.94. The highest mean “index” value was associated with the combination of favorable alleles at *S2A_15755581*, *S4D_481167093*, *S5B_610327124*, and *S6B_616781941*.

### 3.3 Candidate genes, gene ontology, and changes in allele frequency

**Figure 5:**
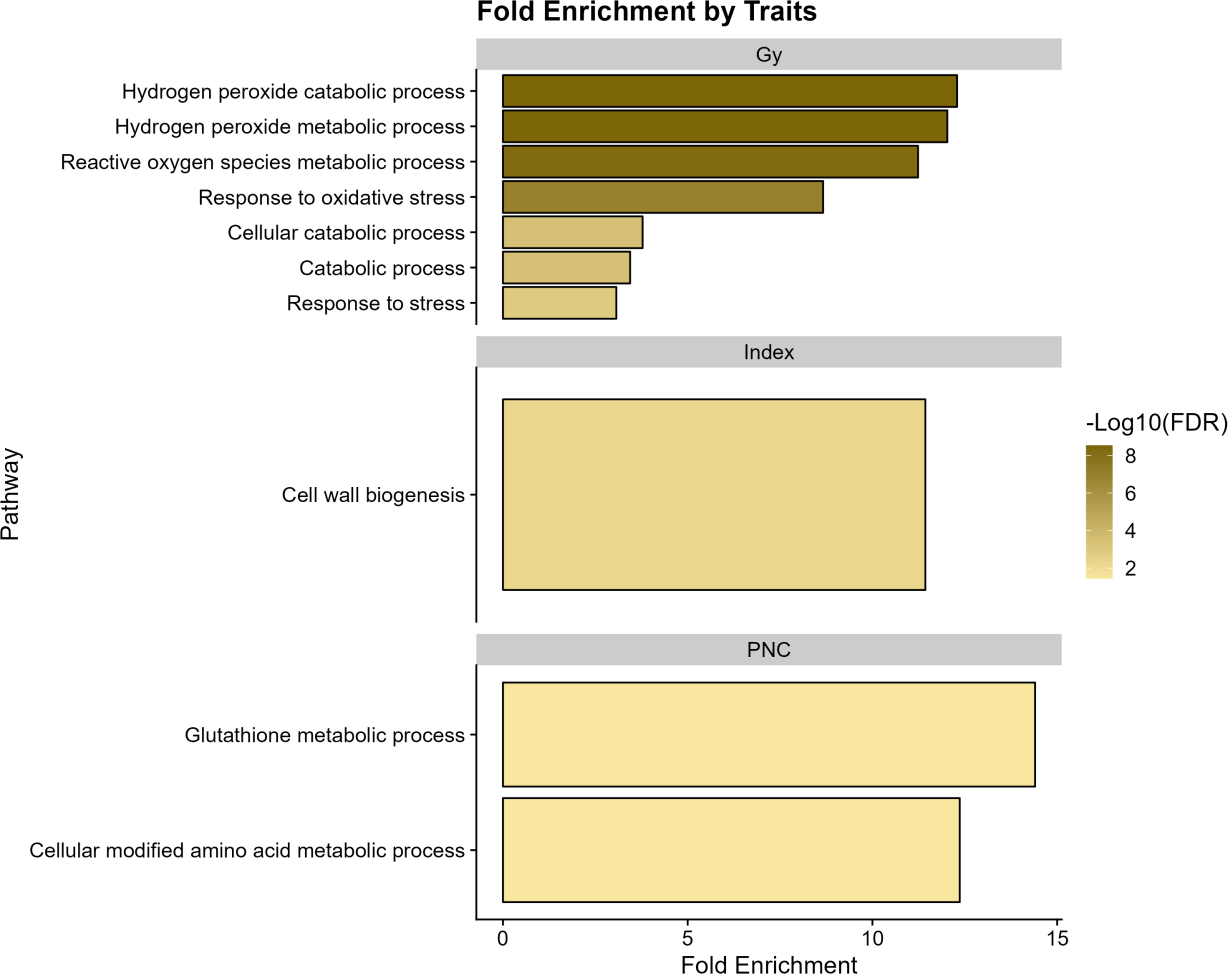
Functional enrichment analysis of genes associated with agronomic variables grain yield (GY), Progeny Number per-Cross (PNC), and “index” in wheat.

The 13 significant markers led to the detection of 430 genes (**Supplementary File 1**). The results of the genetic enrichment analysis for grain yield revealed antioxidant processes, including hydrogen peroxide metabolism, catabolism, and reactive oxygen species, among others (Fold Enrichment > 12). In turn, the “index” traits presented routes related to cell wall biogenesis, whereas for PNC, the most relevant functions were involved in cellular amino acid metabolism and glutathione metabolism (**Figure 5**).

**Figure 6:**
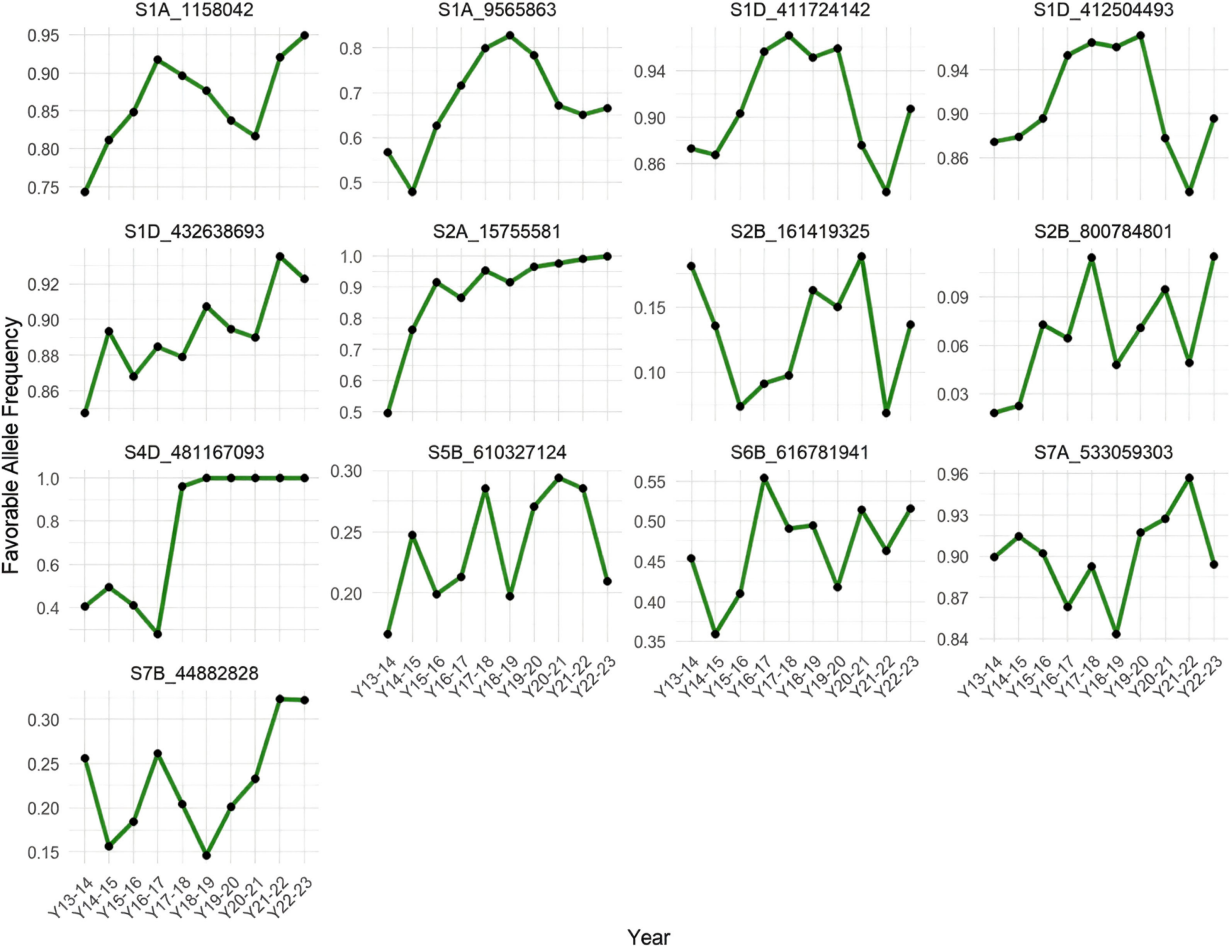
Temporal dynamics of favorable allele frequencies across 10 years in the CIMMYT wheat program (2013–2014 to 2022–2023).

To identify favorable alleles, we examined the allelic effect distributions using boxplots, defining as favorable those alleles associated with increased phenotypic values for key traits. We then tracked the frequency of these favorable alleles across ten breeding cycles, from crop season 2013–2014 to 2022–2023, for the 13 significant MTAs (**Figure 6**).

Our results indicate that several favorable alleles have undergone directional shifts in frequency over time, consistent with positive selection pressure, which is likely applied indirectly by breeders. In particular, markers such as *S1A_1158042*, *S1D_432638693*, *S2A_15755581*, and *S4D_481167093* exhibited marked and sustained increases in favorable allele frequency. These trends suggest that these loci may be linked to traits that have been recurrently targeted by selection, intentionally or unintentionally, during parental advancement and recycling in the CIMMYT wheat breeding program. Notably, the favorable allele at *S4D_481167093* rapidly approached fixation within the population, which may indicate strong selection or linkage to a major-effect gene.

In contrast, other loci (e.g., *S2B_161419325*, *S5B_610327124*, *S7A_533059303*) displayed more erratic patterns in allele frequency, with no consistent directional trend. These irregular fluctuations may reflect genetic drift, the absence of strong selection, or linkage to traits with lower selection intensity or inconsistent value across environments.

### 3.4 Genomic prediction

**Figure 7:**
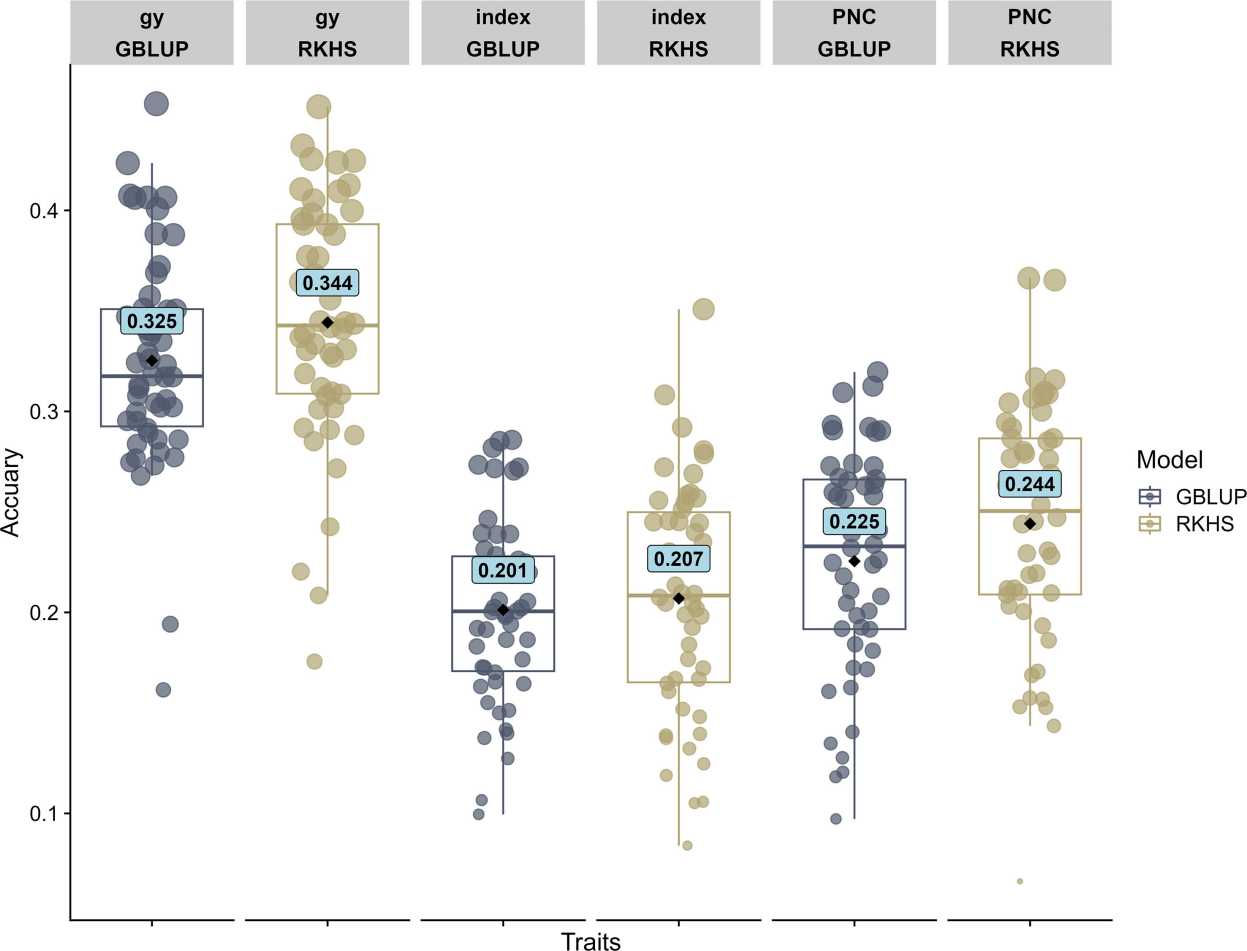
Boxplot of the predictions for the grain yield (GY), Progeny Number per-Cross PNC, and “index” variables using the reproducing kernel Hilber space (RKHS) and genomic best linear unbiased prediction (GBLUP) models.

In general, GY showed a higher prediction accuracy compared to the “index” and PNC for both models that have been used. The results of the genomic prediction analysis demonstrated that the RKHS model showed a slightly better prediction than GBLUP for the variables “index”, GY, and PNC, increasing the prediction accuracy by 3.0%, 5.8%, and 8.3%, respectively. The best predictions were obtained with the RHKS model, with a value of 0.344 for GY, followed by 0.244 for PNC. Finally, the prediction of the “index” showed the lowest accuracy, with a value of 0.207 (**Figure 7**).

## 4 Discussion

Although association studies have been performed for CA in crops such as maize and rice (Chen et al., 2019; Liu et al., 2021; Lu et al., 2020); to date, no GWAS analysis has been reported in the literature for CA-related traits in wheat. Our studies identified eight significant markers associated with grain yield present in chromosomes 1A, 1D, 2B, 7A, and 7B (see **Figure 2**; **Table 1**). Previously, several studies have identified significant markers related to wheat yield on all 21 chromosomes (Akram et al., 2021; Guan et al., 2018; Luján Basile et al., 2019; Pinto et al., 2010; Sukumaran et al., 2015; Turuspekov et al., 2017; Yang et al., 2021). For grain yield, the highest number of significant MTAs occurred on chromosome 1D in positions 411.7, 412.5, and 432.6 Mbp. These locations are close to those reported by Liu (Liu et al., 2020a), which found flanking markers, in wheat for variables associated with yield (*D_contig32020_138–D_GDEEGVY01DD44S_389*; chr1D: 488.6–493.0 Mbp), close to our positions. We similarly identified four MTAs associated with “index” related to yield, located in chromosomes 2A, 4D, 5B y 6D. One of the markers associated with “index” (*S2A_15755581*), was identified near the region described by White et al. (2022), which reported the marker *RAC875_c48625_182* in wheat for grain yield at 2.8 Mbp from our location; this raises an important relationship between the two regions, highlighting the need to study them in more depth. It is also important to note that SNPs *S1D_432638693*, *S7B_44882828*, and *S2A_15755581* were consistently identified by both statistical models (BLINK and FarmCPU). This suggests more confidence in the relevance of these MTAs. Other SPNs associated with “index” and GY, such as *S1A_1158042*, *S1D_432638693*, *S2B_161419325*, *S7A_533059303*, *S7B_44882828*, and *S2A_15755581* require further research due to the limited literature available at this time. In relation to PNC, we identified one MTA (*S1A_9565863*) located on chromosome 1A at position 9.6 Mbp. This finding highlights the importance of continuing studies due to its impact on the selection of elite genotypes.

Additionally, we observed clear differences in grain yield performance across the various FACs (**Figure 3**). The overall mean grain yield of the population was 95.3, whereas the highest-performing FAC reached a mean of 102.58, representing a 7.64% increase relative to the population mean (see Results section). This finding suggests a cumulative additive effect resulting from the combination of specific favorable alleles. Such information is particularly valuable for breeding applications, as it can guide the selection of superior parental combinations. By targeting genotypes that carry optimal FACs, either as general combiners or complementary combiners, breeders can increase the likelihood of producing high-performing progeny.

The utility of FACs has also been documented in previous studies. For example, Wang et al. (2023) identified beneficial FACs for several yield component traits, including kernel number, kernel weight, and thousand-kernel weight, in a panel of 81 wheat varieties. They found that specific FACs could increase kernel weight by 0.34 or 0.26 g per thousand kernels. The strategy of pyramiding favorable alleles with additive effects is not new in wheat breeding and has proven effective for traits with monogenic or oligogenic control, such as grain quality and disease resistance (Liu et al., 2020b; Tyagi et al., 2014).

The results of the genetic enrichment analysis revealed implications of several key biological processes related to the different traits in wheat (see **Figure 5**). The metabolic and catabolic processes of hydrogen peroxide (H_2_O_2_), reactive oxygen species, and oxidative stress response are related to grain yield response. The processes of production and elimination of hydrogen peroxide are involved in plant physiological processes and particularly resistance to stress; however, an excessive accumulation of hydrogen peroxide can activate autophagy in chloroplasts, peroxisomes, and programmed cell death (Quan et al., 2008; Smirnoff and Arnaud, 2019). Similarly, Ullah Zaid et al. (2019) conducted a GWAS analysis to identify MTAs for general CA in rice, focusing on 11 yield-related traits. Their subsequent GO analysis revealed that many of these traits were significantly associated with stress response, metabolic, and biosynthetic processes. We also identified genetic enrichment for indices associated with cell wall biogenesis, highlighting the importance of cell wall functional and structural processes for the development and functionality of the crop (Mehdi et al., 2019). Interestingly, Chen et al. (2021) conducted transcriptome profiling to investigate GCA in barley, with a focus on yield-related traits. Through GO analysis, they identified several differentially expressed genes (DEGs) associated with cellular components, including cell parts and organelles.

On the other hand, PNC showed overexpression of genes in pathways related to the cellular-modified amino acid metabolic process and glutathione metabolic process. The latter plays an important role in biosynthetic pathways, conjugation, and detoxification of xenobiotics as well as reduction of reactive oxygen species (ROS) and is important for better stress tolerance (Dixon et al., 2002; Hasanuzzaman et al., 2019; Noctor et al., 2012; Sahoo et al., 2017).

The analysis of favorable allele frequency dynamics over ten breeding cycles revealed evidence of indirect positive selection acting on several MTAs, notably *S1A_1158042*, *S1D_432638693*, *S2A_15755581*, and *S4D_481167093* (see **Figure 6**). The consistent increase in their frequency suggests that these loci are linked to traits routinely favored during parent and line selection, such as yield or yield-related traits. In particular, the near fixation of some alleles points to strong selection pressure, likely reflecting their importance in breeding progress. In contrast, other MTAs exhibited no consistent temporal trend, possibly due to weak or environment-dependent selection, genetic drift, or association with traits of lower priority.

We acknowledge that GCA in wheat, as in other species, is a highly complex trait. Its genetic basis is governed by the cumulative effects of thousands of small-effect loci, rather than a few major genes (Rojas and Sprague, 1952; Walejko and Russell, 1977). While FACs offer a useful framework for identifying promising complementary crosses, their practical application may be limited by the polygenic nature of the trait. As such, the predictive utility of individual GWAS-derived markers remains constrained in the context of routine selection pipelines. From a genomic prediction perspective, the emphasis shifts from identifying individual significant loci to modeling the entire genomic architecture of the trait. GP approaches, which are based on the infinitesimal model, circumvent the statistical limitations of GWAS by avoiding multiple testing and leveraging genome-wide marker information. While GWAS remains valuable for generating biological insights and identifying occasional candidate regions, its utility for improving complex traits like GCA is limited compared to the predictive accuracy and integration offered by GP models such as GBLUP and RKHS.

Indeed, this is the first study that assessed genomic prediction accuracy for GCA-related traits in wheat. The genomic prediction results showed values ranging from 0.325 and 0.344 for GY, 0.201 and 0.207 for the “index” (see **Figure 7**), similar results were found by Poland and colleagues (2012) for variables associated with wheat yield in a panel of 254 lines, with precisions from 0.28 to 0.45. Our results are also in line with another genomic prediction study in wheat in different environments and years that exhibited prediction accuracies from 0.27 to 0.59 for yield (Gill et al., 2021). The predictive results for PNC had values of 0.225 and 0.244, indicating a moderately limited prediction. In other crops, such as maize, predictions related to GCA ranged from 0.49 to 0.61 (Vélez‐Torres et al., 2018). However, due to the limited information related to the prediction of CA in wheat (Basnet et al., 2019; Zhao et al., 2013), our research could be a starting point for future analyses to improve the prediction of PNC in wheat. Interestingly, our results showed that the RKHS model only slightly outperformed GBLUP for grain yield and other traits, though the differences were not statistically significant. These findings align closely with those reported in durum wheat for grain yield and yield-related traits (Vitale et al., 2024). However, it is important to emphasize that improving prediction accuracy may require integrating diverse data sources and exploring alternative models, such as machine or deep learning (Crossa et al., 2024).

## 5 Conclusions

To date, traits related to combining ability in wheat have not been thoroughly investigated and often fail to produce high-performing offspring despite originating from promising parents. Therefore, a deeper understanding of the combining ability of parental lines is crucial for optimizing the parental selection process. In our study, we identified 13 MTAs, eight of which are related to grain yield (GY), four of which are associated with the “index”, in addition to a marker associated with the number of progeny per cross (PNC). Gene ontology analyses revealed major functions related to hydrogen peroxide metabolism and catabolism, cell wall biogenesis, and amino acid and glutathione metabolism for the traits of interest. Interestingly, several MTAs were found to be under positive selection pressure, indirectly driven by breeders’ decisions over successive cycles. This finding highlights the functional relevance of these loci and reinforces their importance within the wheat breeding pipeline. Likewise, the best predictions were obtained using the RKHS model, with values of 0.344, 0.207, and 0.244 for the traits GY, “index”, and PNC. These findings advance the current understanding of combining ability in wheat, shedding light on MTAs that could serve as valuable tools for developing new markers to better characterize candidate parents. Moreover, this study highlights novel insights into the biological pathways underlying traits related to combining ability. Applying genomic prediction to evaluate the combining ability of candidate parents offers a promising strategy to enhance the efficiency of parental selection. This approach can ultimately improve offspring yield performance and enhance the genetic gain.

## Data Availability

The Hapmap file was share in the figshare repository: https://doi.org/10.6084/m9.figshare.29669330.v1. Additionally, the phenotipic dataset was public shared in the open access repository Figshare (https://doi.org/10.6084/m9.figshare.30024511.v1).
