# Supplementary material for: Unraveling the genetic basis of general combining ability in CIMMYT elite bread wheat germplasm: implications for breeding strategies optimization
Source: Front Plant Sci. 2025 Oct 17;16:1675993. doi: 10.3389/fpls.2025.1675993 (PMC12575131; doi:10.3389/fpls.2025.1675993)
Supplement: Supplementary file 1 [file DataSheet1.docx]

Unraveling the Genetic Basis of General Combining Ability in CIMMYT Elite Bread Wheat Germplasm: Implications for Breeding Strategies Optimization

José I. Saavedra-Ávila^1,2†^, Guillermo S. Gerard^2†^, Salvatore Esposito^3^, Velu Govindan^2^, Julio Huerta-Espino^2,4^, Zerihun Tadesse^2^, Susanne Dreisigacker^2^, Carolina Saint Pierre^2^, Angela Pacheco^2^, Fernando Toledo^2^, Keith A. Gardner^2^, Leonardo Crespo-Herrera^2^, José Crossa^2^*, Paolo Vitale^2^*

^1^Departamento de Genética, Colegio de Postgraduados, Montecillo, Estado de México, Mexico

^2^International Maize and Wheat Improvement Center (CIMMYT), Carretera México-Veracruz Km 45, El Batán, Texcoco, CP, Estado de México, Mexico

^3^Institute of Bioscience and BioResources, National Research Council, Portici, Italy

^4^Campo Experimental Valle de México INIFAP, Chapingo, Edo. de México, Mexico

*** Correspondence:**Corresponding Author
[p.vitale@cgiar.org](mailto:p.vitale@cgiar.org); [j.crossa@outlook.com](mailto:j.crossa@outlook.com)

† These authors contributed equally to this work.


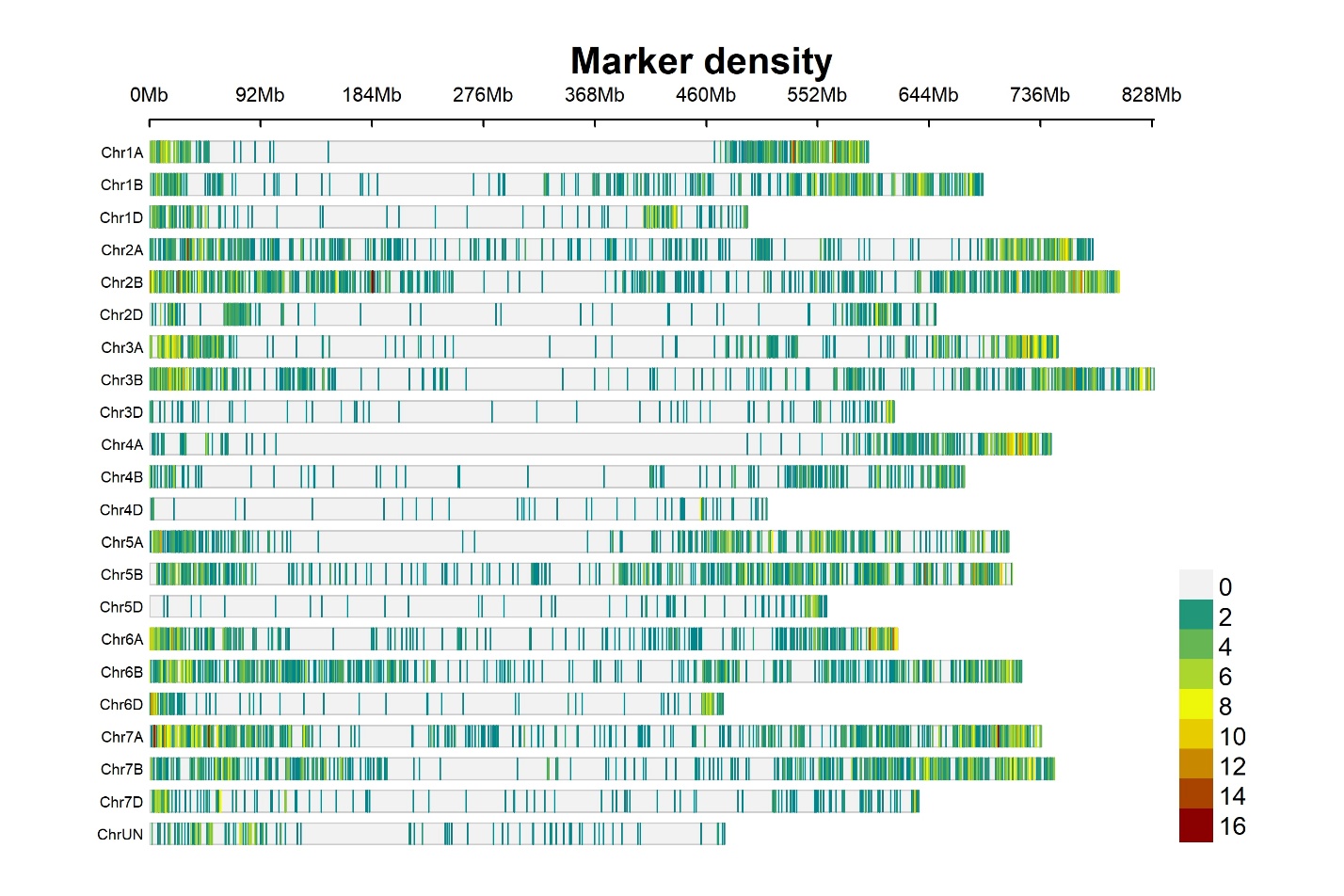


**Figure S1**. Distribution and density of the filtered markers (9890) across the wheat genome. The gradient scale (0-16) indicates regions with high and low SNP density.


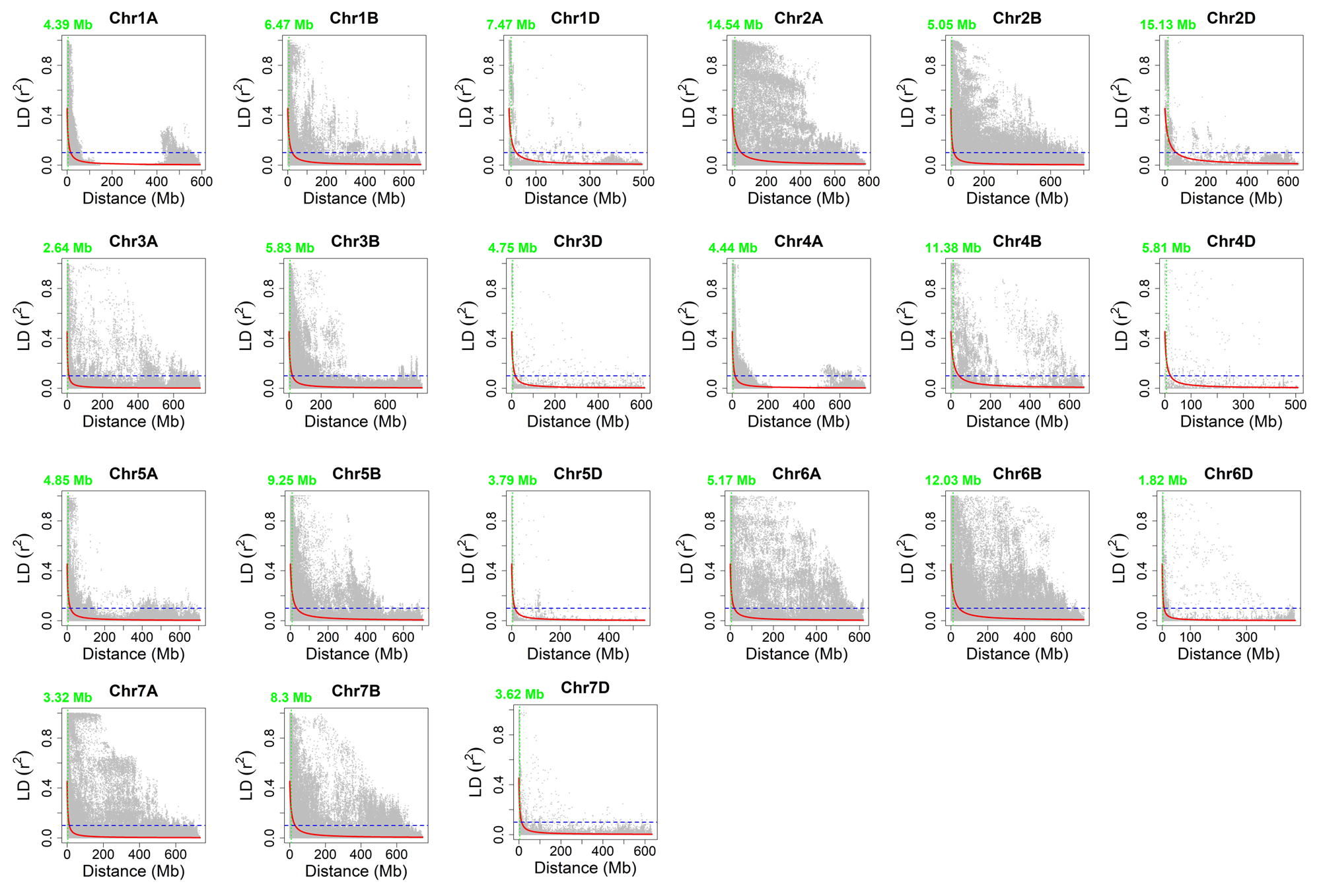


**Figure S2**. Linkage Disequilibrium (LD) decay for each chromosome in the wheat genome


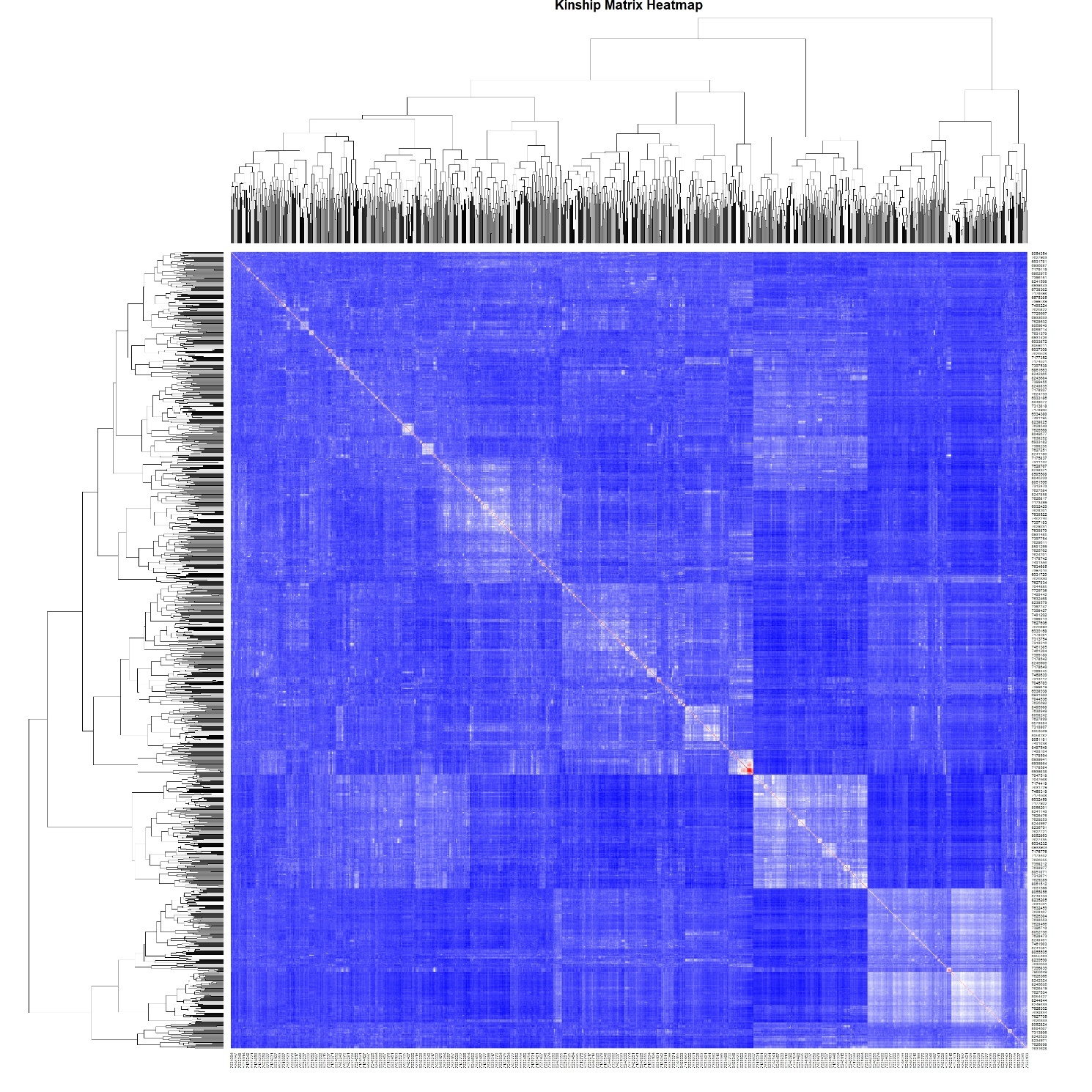


**Figure S3**. Kinship matrix across the genotypes using marker data information


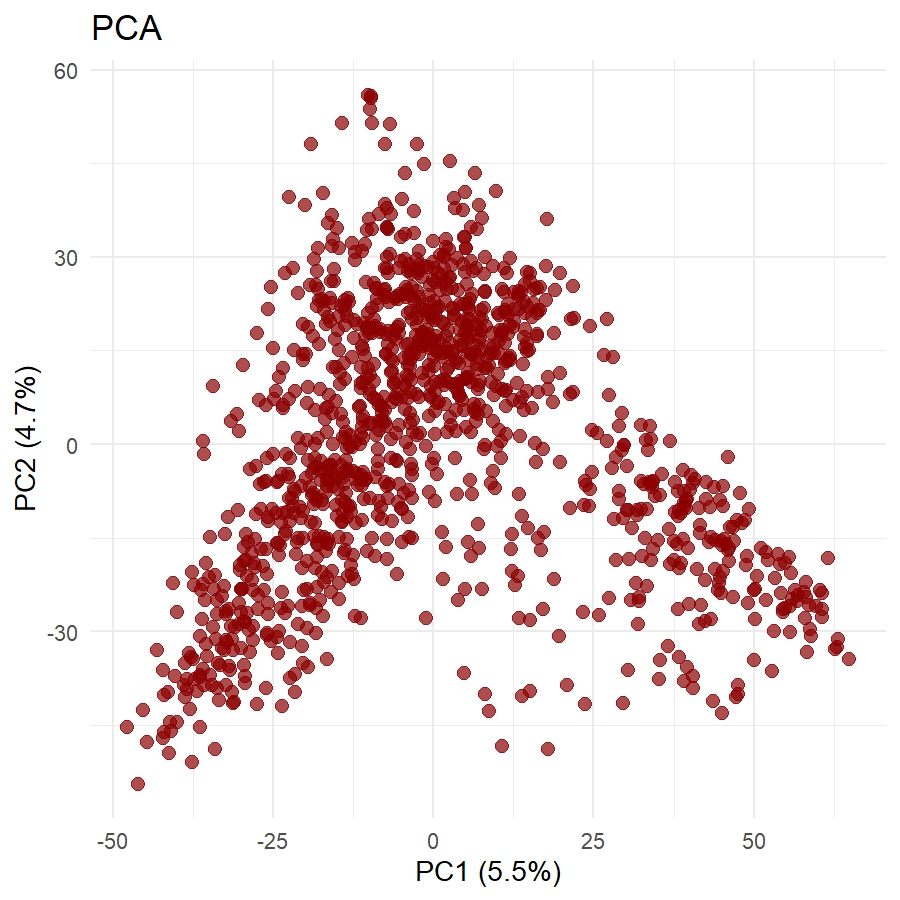


**Figure S4.** Principal Component Analysis (PCA) for all genotypes using molecular markers information.
